# Case Report: Three-dimensional planning for autologous osteoperiosteal transplantation in the treatment of a massive osteochondral defect of the medial femoral condyle

**DOI:** 10.3389/fsurg.2026.1750704

**Published:** 2026-04-10

**Authors:** Yong Xia, Qirui Chen, Ye Ding, Shenghao Cai, Zuorui Zheng, Waleed Khalid, Xiaoling Fu

**Affiliations:** 1 Department of Orthopedics, The Second Affiliated Hospital, Jiangxi Medical College, Nanchang University, Nanchang, Jiangxi, China; 2 Department of Orthopedics, The People's Hospital of Wanzai County, Yichun, Jiangxi, China; 3 Jiangxi Provincial Key Laboratory of Spine and Spinal Cord Disease, Nanchang, Jiangxi, China; 4 Institute of Minimally Invasive Orthopedics, Nanchang University, Nanchang, Jiangxi, China

**Keywords:** 3D planning, autologous osteoperiosteal transplantation, femoral condyle, knee, MASSIVE, osteochondral lesions

## Abstract

**Background:**

Extensive osteochondral defects of the knee, which often cause pain, functional impairment, and potential osteoarthritis, are commonly treated with established strategies such as osteochondral autograft and allograft transplantation. Autologous osteoperiosteal transplantation has emerged as a promising alternative. However, a notable evidence gap exists regarding its application for large osteochondral defects in the knee.

**Case presentation:**

Here, we present a case study of a 21-year-old male farmer with a significant osteochondral defect located on the medial femoral condyle. To achieve a precise match with the anatomical surface geometry of the medial femoral condyle, three-dimensional printing technology was employed to create an autologous osteoperiosteal graft harvested from the ipsilateral iliac crest. This graft was subsequently utilized to repair the large osteochondral lesion. At 22 months postoperatively, the patient exhibited substantial clinical improvement, as evidenced by a Lysholm Knee Score of 90. Follow-up imaging revealed complete integration of the graft with the surrounding bone and restoration of articular congruency. The patient successfully returned to normal activities without experiencing pain or swelling.

**Conclusion:**

This case report demonstrates the successful application of three-dimensional planning for autologous osteoperiosteal transplantation to achieve precise restoration of a massive osteochondral defect in the medial femoral condyle. Favorable postoperative function outcome and good integration of the graft were observed. Therefore, this case provides an alternative therapeutic option for such complex osteochondral defects. Nevertheless, larger studies with long-term follow-up are needed to evaluate the effectiveness and potential complications of this novel technique.

## 1 Introduction

Large osteochondral defects of the knee present significant challenges, often resulting in pain, functional impairment, and progression to osteoarthritis. Osteochondral autograft and allograft transplantation have emerged as reliable treatment options, capable of restoring defects with native hyaline cartilage and subchondral bone in a single surgical procedure (1–3). However, the use of autografts harvested from the femoral condyle is associated with donor-site morbidity, which can range from 0% to 54.5% (4). Moreover, limitations such as the scarcity of donor grafts, contour matching issues, the risk of disease transmission, and high costs restrict the clinical applicability of osteochondral allograft transplantation (5).

Recently, autologous osteoperiosteal transplantation utilizing grafts from the iliac crest has been employed to address osteochondral lesions of the talus (6). Despite the differences in biomechanics and the structure of articular cartilage between the knee and ankle joints, attempts have been made to repair osteochondral lesions in knee cartilage. Lee et al. successfully transplanted an osteoperiosteal autologous iliac crest graft alongside a lateral meniscus allograft to treat osteochondral lesions and a lateral discoid meniscus tear (7).

In this study, we employed both 3D printing technology and autologous osteoperiosteal grafting to address a large osteochondral defect in the medial femoral condyle of a young, active patient, ensuring a precise match to the condylar geometry.

## 2 Case presentation

### 2.1 Clinical data

**Figure 1:**
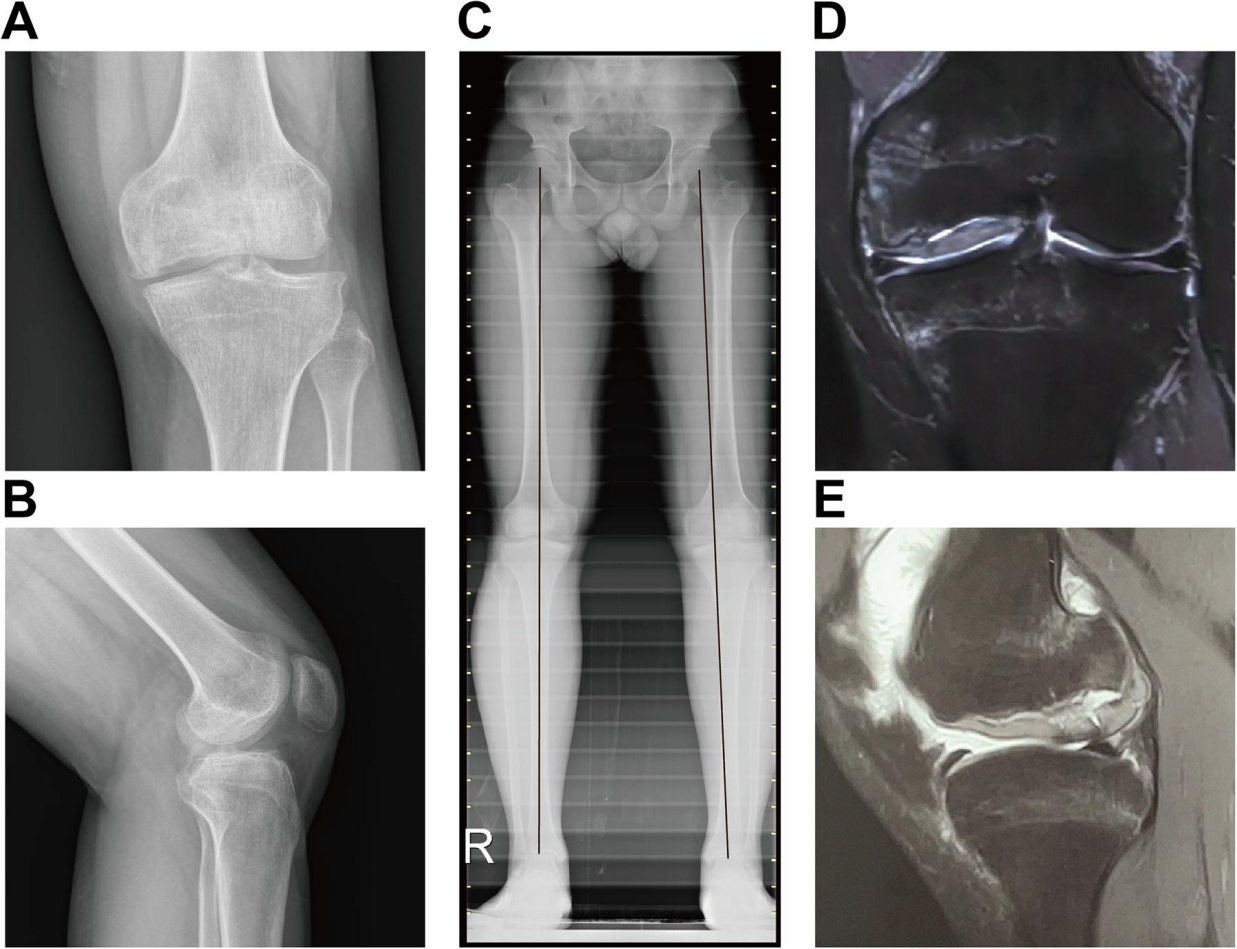
Preoperative x—ray, standing lower extremity radiograph, and magnetic resonance imaging (MRI). **(A,B)** Plain radiographs of the left knee showed an obvious osteochondral lesion with a large bony defect in the MFC and narrowing of the medial joint space. **(C)** Standing lower extremity radiograph showing slight valgus of the left knee. **(D,E)** Preoperative MRI shows the osteochondral lesion in the posterior aspect of the left MFC in the coronal and sagittal planes.

A 21-year-old male farmer was admitted to our hospital in August 2023 due to pain in the left knee. He had a history of knee trauma in 2013 and 2015, although the specifics of these incidents were not documented. Despite undergoing arthroscopic partial meniscectomy and synovectomy at a local hospital in 2023 for suspected meniscal injury and synovial inflammation, his symptoms did not improve, and he experienced significant difficulty in walking. However, detailed operative records were unavailable, and the extent of cartilage damage addressed during the procedure was not documented. The patient was referred to our clinic five months post-arthroscopic surgery. Physical examination revealed tenderness along the medial joint line of the left knee, accompanied by localized swelling. The range of motion (ROM) was restricted to 20°–90°. No ligamentous instability was detected. The patient's pain score was assessed at 8 out of 10, and the Lysholm Knee Score was recorded at 30 out of 100. Plain radiographs of the left knee demonstrated a pronounced osteochondral lesion characterized by a substantial bony defect in the medial femoral condyle (MFC) and narrowing of the medial joint space (Figures 1A,B). Standing alignment of the lower extremities indicated slight valgus of the left knee in comparison to the right knee (Figure 1C). Magnetic resonance imaging of the left knee revealed a significant lesion primarily located in the posteromedial region of the MFC (Figures 1D,E).

### 2.2 Design and fabrication of the prosthesis

**Figure 2:**
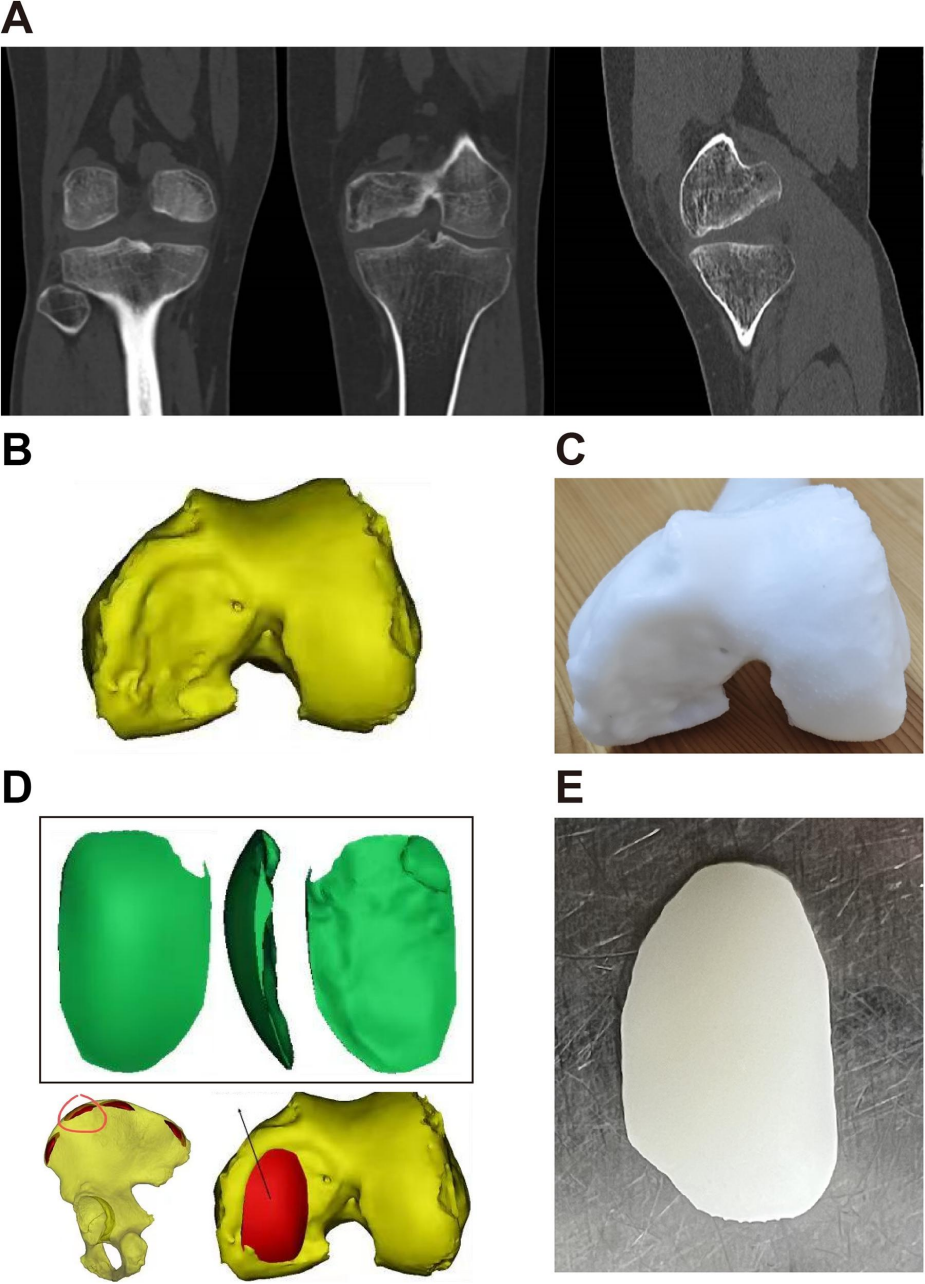
The diagram of a suitable donor site design. **(A)** Preoperative CT scan showing the osteochondral lesion in the posterior aspect of the left MFC. **(B,C)** The three-dimensional printed model of the patient's left knee shows a large osteochondral defect, which is located on the weight-bearing area of the medial femoral condyle and extends posteriorly. **(D,E)** A sterile 3D printed model was designed to screen the location where the curvature of the iliac surface and the exact anatomy fit most for the reconstruction of the osteochondral lesion.

Before the surgery, we performed CT scans of the patient's knee joint and pelvis. The CT images were imported into Mimics software (Materialise, Belgium) for three-dimensional reconstruction. The anatomical models were then processed and exported to 3-matic software (Materialise) for surgical planning and template design, where templates were generated based on the surface morphology of the patient's knee joint and the shape of the cartilage defects (Figures 2A–C). These templates were utilized to identify the most suitable donor site, ensuring that the curvature of the crest surface and the precise anatomy were optimal for osteochondral reconstruction (Figures 2D,E). The physical models were fabricated using a Selective Laser Sintering (SLS) 3D printer (Fuse 1, United States) with Polyamide 12 (PA12) powder, which provides sufficient rigidity for preoperative planning and intraoperative template guidance.

### 2.3 Surgical technique

**Figure 3:**
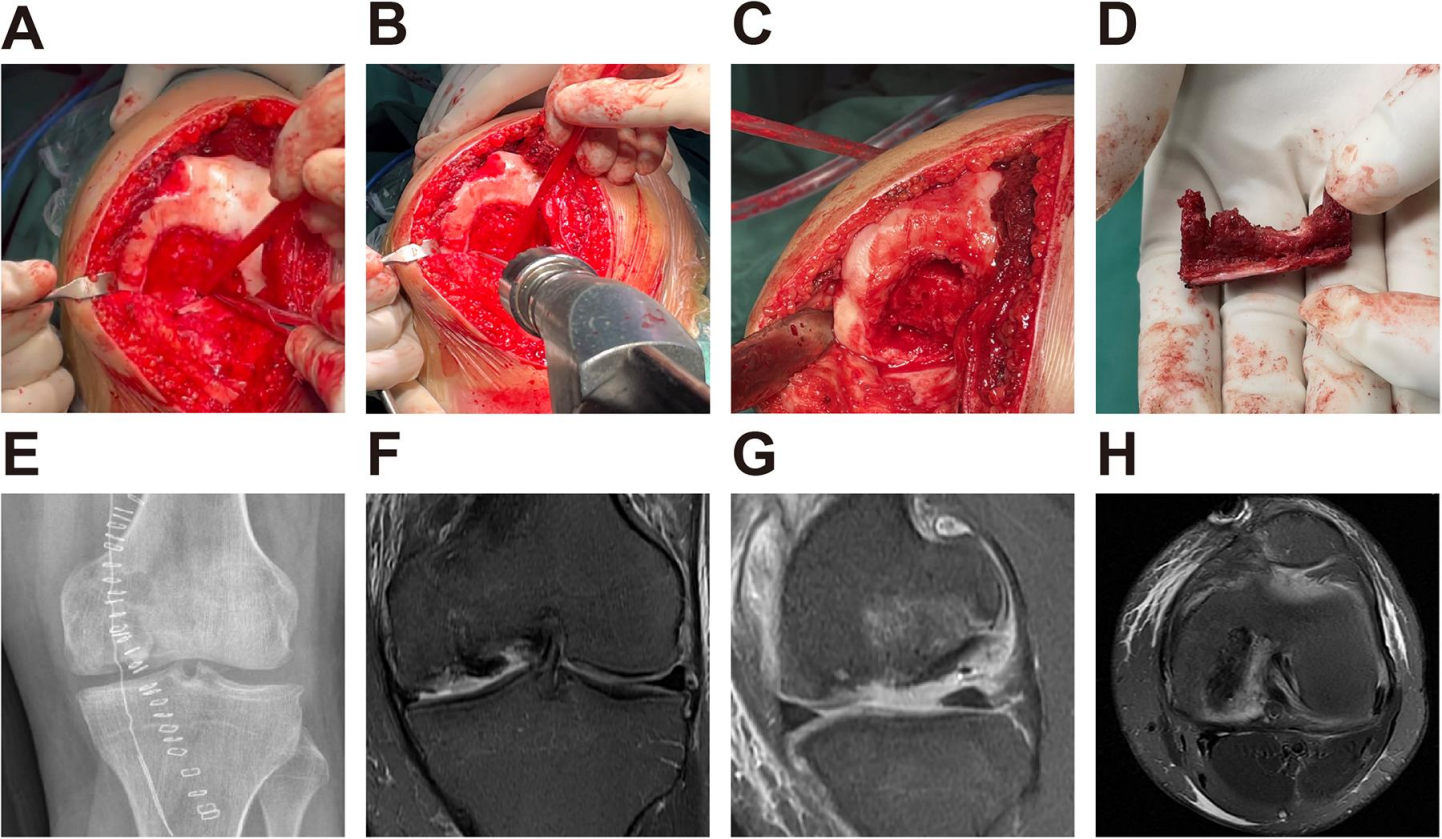
Intraoperative demonstration of autologous osteoperiosteal transplantation and x-ray and MRI after operation at 1 day. **(A)** Photograph of the osteochondral defect on the left medial femoral condyle. **(B)** Multiple drilling using a K-wire was performed toward the debrided lesion. **(C)** Bone sockets were made at the proximal and distal ends of the defect. **(D)** The ends of the graft are modified into protrusions. **(E)** Postoperative x-ray showed that the graft was in a good position. **(F–H)** MRI showed the defect was filled with the osteoperiosteal graft.

The patient was positioned supine, and a medial parapatellar incision was made to expose the joint. The medial and lateral tibiofemoral joints, meniscus, and cruciate ligaments were initially examined. Intraoperative exploration confirmed intact anterior and posterior cruciate ligaments. The lateral meniscus was preserved, whereas the medial meniscus showed partial deficiency from previous surgery. No significant cartilage degeneration was observed in the lateral tibiofemoral compartment. An osteochondral defect was identified on the medial femoral condyle (Figure 3A). The osteochondral lesion was meticulously debrided to remove all unhealthy cartilaginous and fibrous tissues, followed by the use of a 1.5-mm diameter K-wire to penetrate the sclerotic avascular zone (Figure 3B). Bone sockets were created at both the proximal and distal ends of the defect (Figure 3C). Access to the anterior iliac crest was achieved through a 3 cm incision. A sterilized patient-specific 3D-printed template was used intraoperatively to guide graft harvesting. The template was placed on the anterior iliac crest to identify the region with curvature matching the medial femoral condyle defect. The template also defined the precise boundaries and orientation of the graft, allowing accurate osteotomy and minimizing intraoperative estimation. Following this guidance, an osteoperiosteal graft was harvested according to the template. The ends of the graft were shaped into two cylindrical protrusions approximately 4 mm in diameter and 6 mm in length, which were inserted into pre-drilled bone sockets in the femoral condyle to provide press-fit fixation and rotational stability, thereby restoring the lesion. (Figure 3D). The periosteal layer was positioned slightly below the cartilage level. The periosteal surface of the graft was intentionally positioned slightly below the level of the surrounding cartilage to avoid joint surface protrusion and to promote fibrocartilage regeneration. The stability of the graft was assessed during both flexion and extension, and a suction drain was placed in the cavity.

Cefazolin administration continued for 24 h postoperatively, and the suction drain was removed 48 h after the operation. Plain radiography (Figure 3E) and MRI (Figures 3F–H) were performed on the first postoperative day, revealing a well-integrated bone plug. The knee was immobilized in a cast, and ROM exercises were not permitted until 3 weeks postoperatively. Weight-bearing was restricted for the initial 6 weeks. Partial weight-bearing was allowed after 6 weeks, and full weight-bearing was achieved at approximately 12 weeks postoperatively, depending on clinical symptoms and radiographic evaluation.

### 2.4 Postoperative management and follow-up

**Figure 4:**
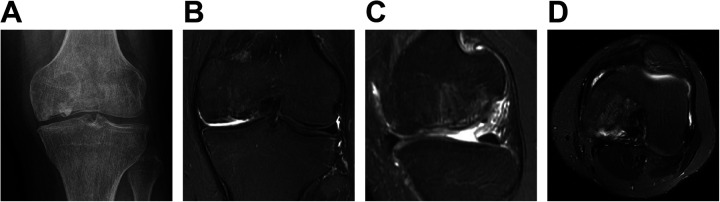
x-ray and MRI after operation at 22 months follow-up. **(A)** Follow-up x-ray showed no stenosis of the joint space. **(B–D)** Complete integration of the graft with the bone and articular congruency was observed in MRI.

During the most recent follow-up, which occurred 22 months after the surgical procedure, the patient exhibited a satisfactory recovery from the surgical wound, accompanied by a substantial alleviation of pain. The patient demonstrated significant clinical improvement with a Lysholm Knee Score of 90. Complete integration of the graft with the bone and articular congruency was observed in follow-up imaging (Figure 4). The patient successfully performed all activities of daily living and farm work.

## 3 Discussion

Autologous osteoperiosteal transplantation harvests autografts from the iliac crest with the overlying periosteal layer. The periosteum has been demonstrated to have a good ability for cartilage regeneration (8–11). Similar to autologous or allograft osteochondral transplantation, this technique can simultaneously repair both cartilage and subchondral bone, which has been proposed as a treatment option for OCDs in the ankle joint (12). Many studies have shown satisfactory clinical outcomes of this procedure (13, 14). Shi et al.'s retrospective cohort study demonstrated that autologous osteoperiosteal transplantation exhibited comparable clinical efficacy to osteochondral transplantation, while concomitantly exhibiting a reduced incidence of donor-site morbidity (15). In contrast to the limited sources of osteochondral grafts, the source of osteoperiosteal grafts that were harvested from the iliac crest is very abundant (16). Matrix autologous chondrocyte implantation is another alternative for osteochondral defects, but the disadvantages, including 2-stage treatment and substantial costs, hinder the clinical application (17, 18). Compared with current techniques, autologous osteoperiosteal transplantation has the advantages of effective clinical effects, one-stage operation, low cost, abundant source of graft, and donor-site morbidity. However, Most of them focused on repairing articular cartilage damage in the talus. Recently, Lee et al. successfully applied this technique to the repair of cartilage injury on the femoral condyle (7). Yang et al. investigated the effect of osteoperiosteal autografts on integrated osteochondral repair in a large animal model. The results showed that it has a promising repair effect and is being increasingly adopted in clinical practice (19).

Therefore, autologous osteoperiosteal transplantation was applied to repair the large osteochondral defect in our case. Keeping consistent congruency between the implanted grafts and the surrounding native articular surface is very important for clinical outcomes. Naked-eye manual harvesting, which is limited by individual surgeon experience and ability, can not determine the ideal size and shape of the graft to match the complex curvature of the femoral condyle surface. Three-dimensional printing technology can objectively quantify exact individual parameters and has rapidly gained clinical acceptance in reconstructive surgery (20, 21). Several studies have demonstrated the utility of 3D printing in osteochondral reconstruction, particularly in osteochondral allograft transplantation, where donor-recipient curvature matching is essential for restoring joint congruity (22–24). However, most previous studies focused on osteochondral grafts rather than osteoperiosteal grafts. In contrast, our approach applies 3D curvature matching specifically to autologous osteoperiosteal graft harvesting from the iliac crest, which has rarely been described in the literature. This donor-site-focused application of 3D technology for osteoperiosteal grafts represents a novel contribution to the field. In our particular case, the 3D template was used to screen for the location where the curvature of the iliac crest fit most for MFC reconstruction and mark the resection line for harvesting graft extremely accurately. The postoperative imaging data showed that the defect and the shape of the medial femoral condyle were well reconstructed. The patient fully recovered his normal work and life at the last follow-up. In the future, patient-specific instrument (PSI) guides based on 3D planning may further enhance surgical precision and reduce operative time during osteoperiosteal graft harvesting.

## 4 Conclusion

Despite the limitations associated with short-term follow-up and the limited number of cases, 3D planning for autologous osteoperiosteal transplantation demonstrates significant advantages in reconstructing both the morphology and function of the knee. This technique represents an innovative, viable, and efficient surgical option for patients suffering from complex, large cartilage defects, particularly for young patients with high functional demands. It not only effectively alleviates patient pain and enhances quality of life but also decelerates the progression of osteoarthritis in the knee, thereby opening new avenues for the treatment of cartilage defects. However, to further enhance the representativeness and generalizability of the findings and to promote the clinical application of this technique, it is essential to expand both the sample size and diversity through multicenter studies.

## Data Availability

The original contributions presented in the study are included in the article/Supplementary Material, further inquiries can be directed to the corresponding author.
